# Immune system and angiogenesis-related potential surrogate biomarkers of response to everolimus-based treatment in hormone receptor-positive breast cancer: an exploratory study

**DOI:** 10.1007/s10549-020-05856-3

**Published:** 2020-08-07

**Authors:** Francesco Schettini, Navid Sobhani, Anna Ianza, Tiziana Triulzi, Alfredo Molteni, Maria Chiara Lazzari, Carla Strina, Manuela Milani, Silvia Paola Corona, Marianna Sirico, Ottavia Bernocchi, Fabiola Giudici, Maria Rosaria Cappelletti, Eva Ciruelos, Guy Jerusalem, Sherine Loi, Stephen B. Fox, Daniele Generali

**Affiliations:** 1 Department of Clinical Medicine and Surgery, University of Naples Federico II, Naples, Italy; 2 Translational Genomics and Targeted Therapeutics in Solid Tumors, August Pi I Sunyer Biomedical Research Institute (IDIBAPS), Barcelona, Spain; 3 SOLTI Breast Cancer Research Group, Barcelona, Spain; 4 Department of Medical, Surgery & Health Sciences, University of Trieste, Piazza Ospitale 1, 34129 Trieste, Italy; 5 Molecular Targeting Unit, Department of Research, Fondazione IRCCS Istituto Nazionale dei Tumori, Milan, Italy; 6 UO Ematologia e CTMO, ASST di Cremona, Viale Concordia 1, 26100 Cremona, Italy; 7 UO Multidisciplinare di Patologia Mammaria e Ricerca Traslazionale, ASST di Cremona, Viale Concordia 1, 26100 Cremona, Italy; 8 Department of Medical Oncology, Breast Cancer Unit, University Hospital, 12 de Octubre, Avda de Córdoba s/n, Madrid, Spain; 9 Department of Medical Oncology, Centre Hospitalier Universitaire de Liège and Liège University, Avenue de L’Hòpital 1, 4000 Liège, Belgium; 10 Peter MacCallum Cancer Center, 305 Grattan Street, Melbourne, VIC Australia; 11 University of Melbourne, Melbourne, VIC Australia

**Keywords:** Hormone receptors, Breast cancer, Everolimus, mTOR, Biomarker, Immunomodulation

## Abstract

**Purpose:**

mTOR inhibitor everolimus is used for hormone receptor-positive (HR+)/HER2-negative metastatic breast cancer (mBC). No reliable predictive biomarker of response is available. Following evidences from other solid tumors, we aimed to assess the association between treatment-associated immune system features and everolimus activity.

**Methods:**

We retrospectively explored a correlation with the therapeutic activity of everolimus and tumor-associated immune pathways with ingenuity pathway analysis (IPA), neutrophil-to-lymphocyte ratio (NLR), circulating lymphocytes, and endothelial cells (CECs) in 3 different HR+ mBC studies, including the BALLET phase IIIb study.

**Results:**

The circulating levels of CD3^+^/CD8^+^, CD3^+^/CD4^+^, and overall T lymphocytes were higher in responders versus non-responders at baseline (*p* = 0.017, *p* < 0.001, *p* = 0.034) and after treatment (*p* = 0.01, *p* = 0.003, *p* = 0.023). Reduced CECs, a tumor neoangiogenesis marker, were observed in responders after treatment (*p* < 0.001). Patients with low NLR (≤ 4.4) showed a better progression-free survival compared to patients with high NLR (> 4.4) (*p* = 0.01). IPA showed that the majority of immunity-related genes were found upregulated in responders compared to non-responders before treatment, but not after.

**Conclusions:**

Lymphocytes subpopulations, CECs and NLR could be interesting biomarkers predictive of response to everolimus-based regimens, potentially useful in daily clinical practice to select/monitor everolimus-based treatment in mBC. Further studies to confirm such hypotheses are warranted.

## Introduction

Despite the demonstrated efficacy of anti-hormonal treatment in patients with hormone receptor-positive (HR+) breast cancer (BC), intrinsic and acquired endocrine resistance occurs in a significant proportion of patients, leaving this tumor being still one of the most common causes of cancer-related death in women [1, 2]. One mechanism of resistance relies on mTOR, a downstream effector of the phosphatidylinositol-3-kinase (PI3K) pathway, which is implicated in cell growth and survival, angiogenesis, and immune regulation [3]. The PI3K/Akt/mTOR pathway frequently contributes to breast cancer progression playing a central role in multiple cellular functions and is a key mechanism of resistance to endocrine therapy [2, 3]. The mTOR inhibitor everolimus is approved for HR+/HER2-negative (−) locally advanced or metastatic BC (mBC) treatment in combination with the aromatase inhibitor (AI) exemestane [4]. However, benefit from everolimus is variable and reliable biomarkers for the selection of patients who will most likely respond are urgently needed [5].

There has been accumulating evidence suggesting that the efficacy of conventional anticancer therapies might rely, at least in part, on eliciting an anti-tumor immune response [6, 7]. In fact, several conventional chemotherapeutics, as well as targeted anticancer drugs, seem to modify the composition and activity of the tumor infiltrate, affecting treatment efficacy and ultimately outcome [6, 7]. Moreover, the local or systemic immune system in patients with cancer appears to be of prognostic value and might be used to predict the therapeutic response to specific treatments [8]. Furthermore, recent evidence concerning the efficacy of immune-checkpoint inhibitors in PD-L1 positive triple negative (TN) BC has recently reignited the interests in BC immunotherapy and highlighted the potentially relevant role of immune modulation in BC treatment [9–11].

Everolimus acts by blocking cell growth and metabolism; it is a powerful immune-suppressor used to avoid organ rejection in renal transplanted patients [12, 13] by controlling homeostasis and the balance between effector T cells and regulatory T cells (Tregs) [14]. There is also emerging evidence highlighting the immunomodulatory role of everolimus in solid tumors such as renal cell [8, 15, 16] and hepatocellular carcinoma [17]. To the best of our knowledge, no data are available about the role of mTOR axis inhibitors on the immune system in BC treatment.

Based on preliminary evidence regarding everolimus immunomodulatory role in several solid tumors [8, 15–17], we have investigated immune infiltrate and circulating immune cells in BC using several cohorts of patients treated with everolimus. Firstly, we obtained tumor biopsies and circulating lymphocytes populations in blood samples from patients with mBC to explore for potential differences among everolimus responders vs. non-responders. Secondly, we investigated a potential correlation between neutrophils-to-lymphocytes ratio (NLR) and progression-free survival (PFS) in the BALLET trial [18] and, thirdly, we performed differential gene immune expression analyses between everolimus responders and non-responders on tissue samples from a window-of-opportunity trial in locally advanced breast tumors. Finally, in blood samples from mBC patients we also investigated the potential presence of different levels of circulating endothelial cells (CECs) between everolimus responders and non-responders. The amount of circulating CECs correlates with angiogenesis in cancer and seem to correlate with plasma levels of angiogenic mediators VCAM-1 and VEGF [19, 20], many of whose downstream pathways are also inhibited by everolimus, thus being a potential biomarker of its activity.

Overall, the aim of our study was to preliminarily find out potential easy-to-detect biomarkers of response related to immune system and neoangiogenesis, to better selecting patients that may benefit from everolimus-based therapy.

## Materials and methods

### Case selection and studies descriptions

In our analysis, we retrospectively included postmenopausal patients affected by locally advanced or metastatic HR+ BC treated with everolimus-based regimens in 3 previous different clinical studies. Patients came from three separate cohorts pertaining to the MREC trial, the mTOR Study and the BALLET trial.

The first one was a window-of-opportunity trial based on the administration of 5 mg everolimus in neoadjuvant locally advanced setting for 14 days prior to surgery. The study enrolled 32 women diagnosed with operable HR+ BC. Study details and population demographics have been previously reported [21].

The mTOR Study was a prospective trial enrolling a total of 15 consecutive postmenopausal women diagnosed with relapsed HR+/HER2− mBC, treated in the first-line setting at the ASST-Cremona (Italy) with 10 mg of everolimus alone daily for 21 days, followed by the combination with exemestane (25 mg) until progression. Patients had relapsed after primary tumor surgery and adjuvant endocrine therapy with a non-steroidal aromatase inhibitor administered for 5 years. Pathologists from the ASST-Cremona performed all the histopathological diagnoses. Tissue samples were collected from the most accessible metastatic site in order to perform immunohistochemical (IHC) analysis before everolimus single agent administration and after 21 days, before the addition of exemestane; clinical data were retrieved from patients’ charts in the Breast Unit of the ASST-Cremona. Blood samples were also obtained from patients enrolled before and after everolimus administration, for flow cytometry analysis. Responsiveness to everolimus was measured by ^18^FDG-PET/CT after 21 days of everolimus-based treatment, at the 3^rd^ month and every 3 months until progression. Patients were considered responsive to everolimus when a reduction of SUV_max_ was present at first 21 days and maintained for the first 9 months at least; whereas with a detection of increase or stability in SUV_max_ during the 9th months of treatment, the patients were classified as non-responsive.

The BALLET study was an expanded access European, phase IIIb, open-label, single-arm, multicenter clinical trial (EudraCT Number: 2012-000073-23), which has been previously described [18].

### Immunohistochemistry

Tissue from tumor specimens was obtained through biopsy of the metastasis of 15 patients with mBC within the mTOR Study, embedded in paraffin and fixed in formalin (FFPE) for IHC analysis. Regions with non-invasive carcinoma, normal tissue, or necrosis were excluded from the evaluation. Standard IHC was performed on FFPE for HER2, estrogen receptor (ER), progesterone receptor (PgR), and Ki67 and CD31 staining using standard protocols as described elsewhere [22–25]. Considering a demonstrated performance of circulating endothelial cells (CECs) and CD31 expression as a biomarker mirroring the occurrence of angiogenesis in the tumor [19], and given that PI3K/mTOR pathway is involved in angiogenesis, we also evaluated patients’ CECs and CD31 modulation before/after treatment as a measure of everolimus’ on-target activity.

### Flow cytometry analysis

The study of circulating immune cells and CECs was performed on samples coming from the mTOR Study. The whole blood samples before and after treatment allowed to analyze circulating cells and their changes under therapy. Flow cytometry analysis was performed with dual or triple-laser flow cytometers Becton Dickinson (BD) FACSCanto™ and BD FACSCanto II™, with BD™ Cytometer Setup and Tracking (CS&T) control, in order to make the signals reproducible and comparable regardless of the variation in environmental conditions. Acquisition of at least 1.5 × 10^6^ events was assessed by BDFACSC Diva software. The lymphocytes subpopulations (B, NK, T with CD4 and CD8 subpopulation) were assessed with BD Multitest 6-Color TBNK kit (Becton Dickinson™). The kit contains FITC-labeled CD3 (SK7clone), PE-labeled CD16 (B73.1 clone) and CD56 (NCAM 16.2 clone), CD45 (2D1 clone) conjugated with the fluorochromes PerCP-Cy5.5, CD4 (SK3 clone) conjugated with PE-Cy7, and CD19 (SJ2SC1 clone) conjugated with APC and CD8 (SK1 clone) conjugated with APC-Cy7. The BD FACSCanto clinical software was employed to carry out the analysis. Leucocytes were identified by CD45 expression and SSC/FCS morphological parameters. T lymphocytes were sorted by CD3 expression and then split into CD4 and CD8 populations. CD3 negative cells were split into B lymphocyte (expressing CD19) and NK cells (CD16 and CD56 positive). Subpopulations absolute count was done by the “trucount tube” (BD™) containing a known number of beads. The T-reg cells (CD4 positive, bright CD25 positive and CD127 negative) were sorted using single Becton Dickinson monoclonal antibodies: CD3 (SK7 clone) conjugated with the fluorochromes FITC, CD25 (2A3 clone) conjugated with PE, CD4 (SK3 clone) conjugated with PerCP-Cy5.5 and CD127 (HIL-7R-M21 clone) conjugated with V450, and CD45 (HI30 clone) conjugated with V500.

The CECs are uncommon findings in the peripheral blood. They can be identified by CD45 negativity with CD31 and CD146 positivity. CECs sorting was assessed using a three-color panel: CD31 (WM59 clone) conjugated with the fluorochromes FITC, CD146 (P1H12 clone) conjugated with PE, and CD45 (2D1 clone) conjugated PerCP-Cy 5.5.

### Gene expression and statistical analyses

The gene expression data used in this study were derived from the population of the MREC Study [21, 26]. Microarray data were processed starting from the authors’ raw data. Class comparison analysis was performed using the Bioconductor package [27]. The probes from Illumina profile expression data were normalized using quantile normalization within the beadarray package and batch processing effects were corrected using the combat tool [28, 29]. Pairwise Significance Analysis of Microarrays (SAM) implemented with siggenes package was used to identify the differentially expressed genes and to predict false discovery rate (FDR) [30]. To define significantly differentially expressed genes, an FDR < 5% was applied as cutoff. The data on the reduction in the percentage of Ki67-positive cells after treatment were used to separate responders from non-responders. Analyses were performed using R, version 3.4.2, and BioConductor, release 3.6 [27, 31]. We used the list of differentially expressed genes to analyze our patients’ cohorts for enrichment in canonical signaling pathways, in order to evaluate potential enrichment in immune pathways through ingenuity pathway analysis (IPA) [32]. The web-based pathway analysis tool QIAGEN IPA (QIAGEN Digital Insights, https://digitalinsights.qiagen.com) was used. Patients were separated into 2 groups according to response to everolimus neoadjuvant treatment as illustrated in a previously published work [21] and IPA on differentially expressed genes between these 2 groups was performed at two different time points (i.e., before and after therapy completion).

Circulating immune cells and CECs, median levels in blood were calculated with standard non-parametric statistical methods (Mann–Whitney test for unpaired data, Wilcoxon’s matched-pairs signed-rank test for paired data, Spearman Rho for simple correlation analysis). Statistical analyses were performed using the Statistica software (Statsoft, Tulsa, OK, USA) for Windows (Microsoft, Redmond, WA, USA) software.

A post hoc analysis was conducted from the neutrophils and lymphocytes values were derived from the BALLET study in order to investigate a correlation with survivals of patients. Information about the neutrophil and lymphocyte status was collected at basal and at the time of progression from the combination of everolimus/exemestane, when available. NLRs were calculated based on four cutoff values and patients discriminated based on four quartiles according to Santoni et al. [15]. NLR was calculated by dividing the absolute neutrophil count by the absolute lymphocyte count. Pre-treatment percentage of neutrophils and NLRs was considered. The Kaplan–Meier method was used to assess PFS differences according to NLRs, and the log-rank test was used to evaluate the significance of each comparison. PFS was defined as the time from the first day of study treatment until disease progression or death, whichever occurred first.

The analyses were conducted on SPSS (15.0 version; SPSS Inc., Chicago, IL, USA). All analyses were two-sided and statistical significance was established at the *p* < 0.05 level. REMARK criteria were followed to report data [33].

## Results

### Circulating immune-related cells and CECs in patients according to response to everolimus in the metastatic setting

**Fig. 1:**
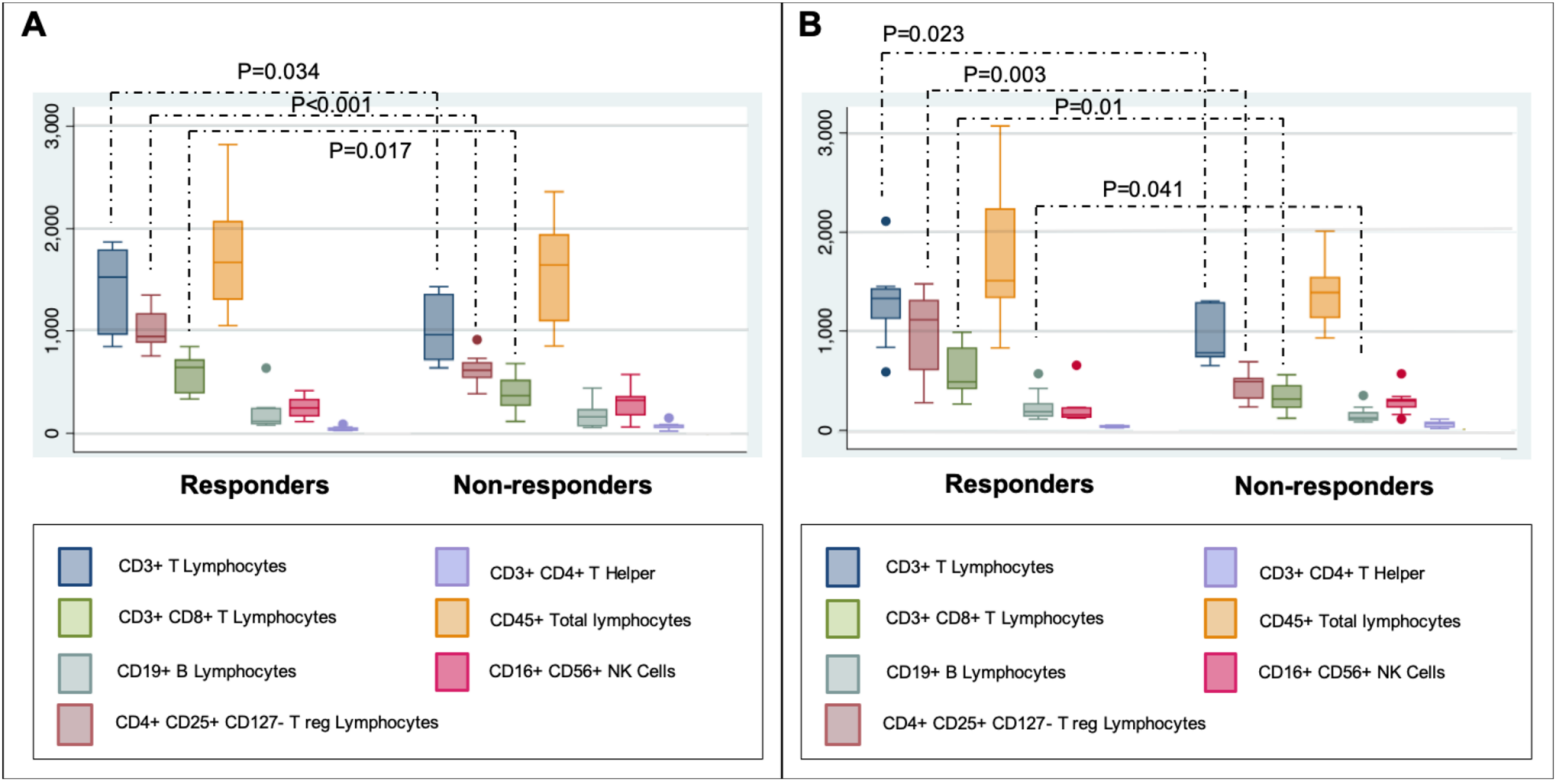
Quantification of lymphocytes populations in the blood of responders and non-responders at basal (**a**) or after (**b**) everolimus therapy. Only significant p-values from unpaired t-test are reported

**Fig. 2:**
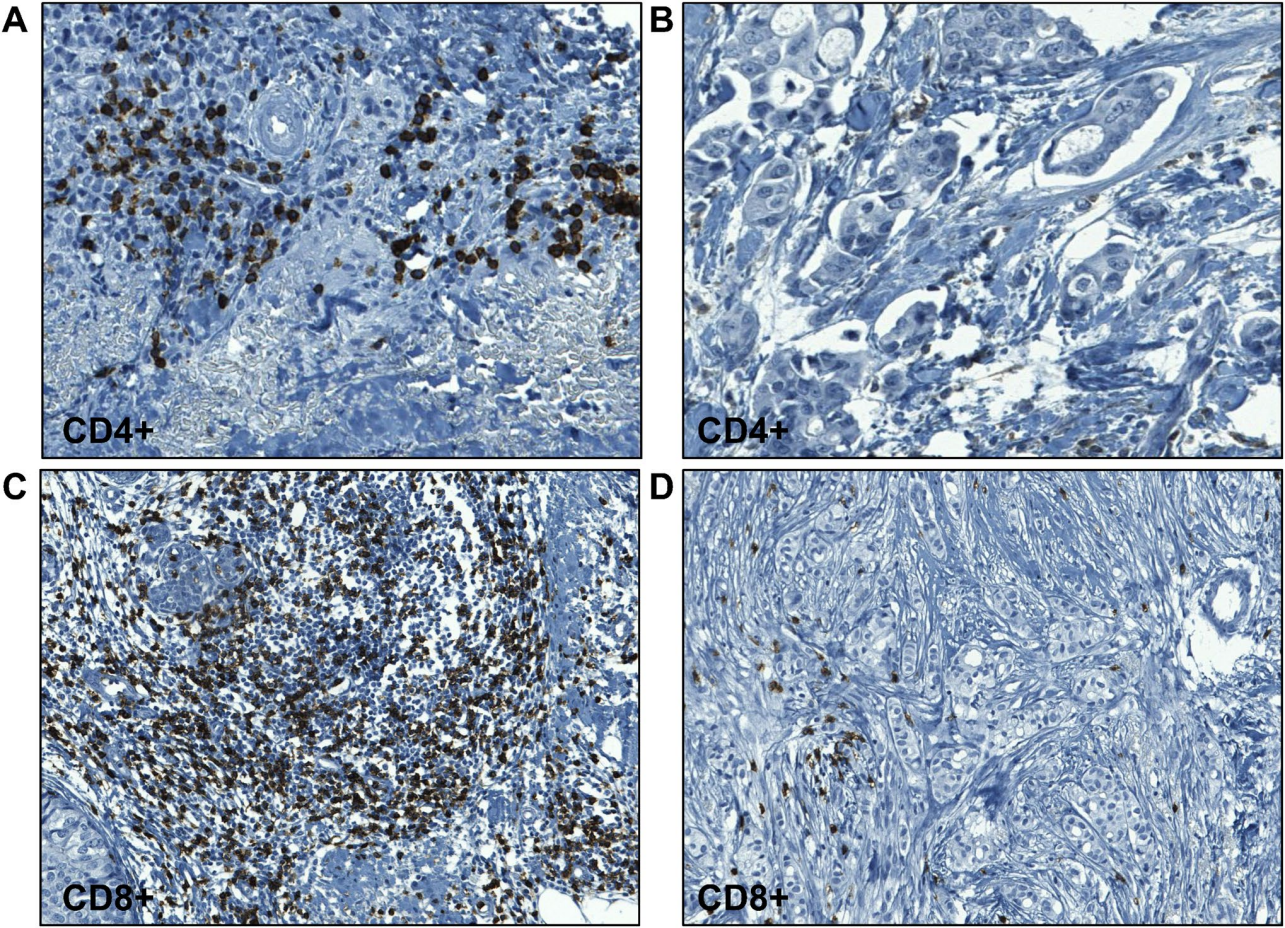
Representative images of CD3^+^/CD4^+^ T cells (**a**, **b**) and CD3^+^/CD8^+^ T cells (**c**, **d**) infiltrating tumor tissues of responsive (**a**–**c**) and non-responsive (**b**–**d**) patients

Based on the association between expression of immune-related genes in tumors responsive to short-term everolimus in neoadjuvant setting, we investigated whether the number of circulating immune cells could predict response to 10 mg everolimus administered alone in a cohort of 15 patients with mBC (Fig. 1a, b). While no difference in the number of CD45^+^ total lymphocytes at baseline or after treatment was found between responders and non-responders, the levels of CD3^+^ T lymphocyte were higher in responders versus non-responders at both baseline (*p* = 0.034) and after treatment (*p* = 0.023)*.* Likewise, the levels of T lymphocytes CD3^+^/CD8^+^ and CD3^+^/CD4^+^ were higher in responders compared to non-responders at baseline (*p* = 0.017, *p* < 0.001, respectively) and after treatment (*p* = 0.01, *p* = 0.003, respectively). In contrast, there was no statistically significant difference in the number of CD19^+^ B-lymphocytes between responders and non-responders at both baseline and final stages of treatment. There was a trend of a reduced number of T-regulatory lymphocytes CD4^+^/CD25^+^/CD127^−^ in responders compared with non-responders at baseline (*p* = 0.075) and post-treatment (*p* = 0.059), although not statistically significant. CD16^+^/CD56^+^ NK cells showed no difference in number at baseline, but responsive tumors post-treatment showed slightly lower circulating NK cells compared with non-responders (*p* = 0.041). Interestingly, the higher number of circulating CD4+ and CD8+ T cells was associated with higher pre-treatment infiltration of these cells in the tumor microenvironment of responsive patients (Fig. 2a–c) compared to non-responders (Fig. 2b–d), as evaluated by IHC in both primary and metastatic lesions.

**Fig. 3:**
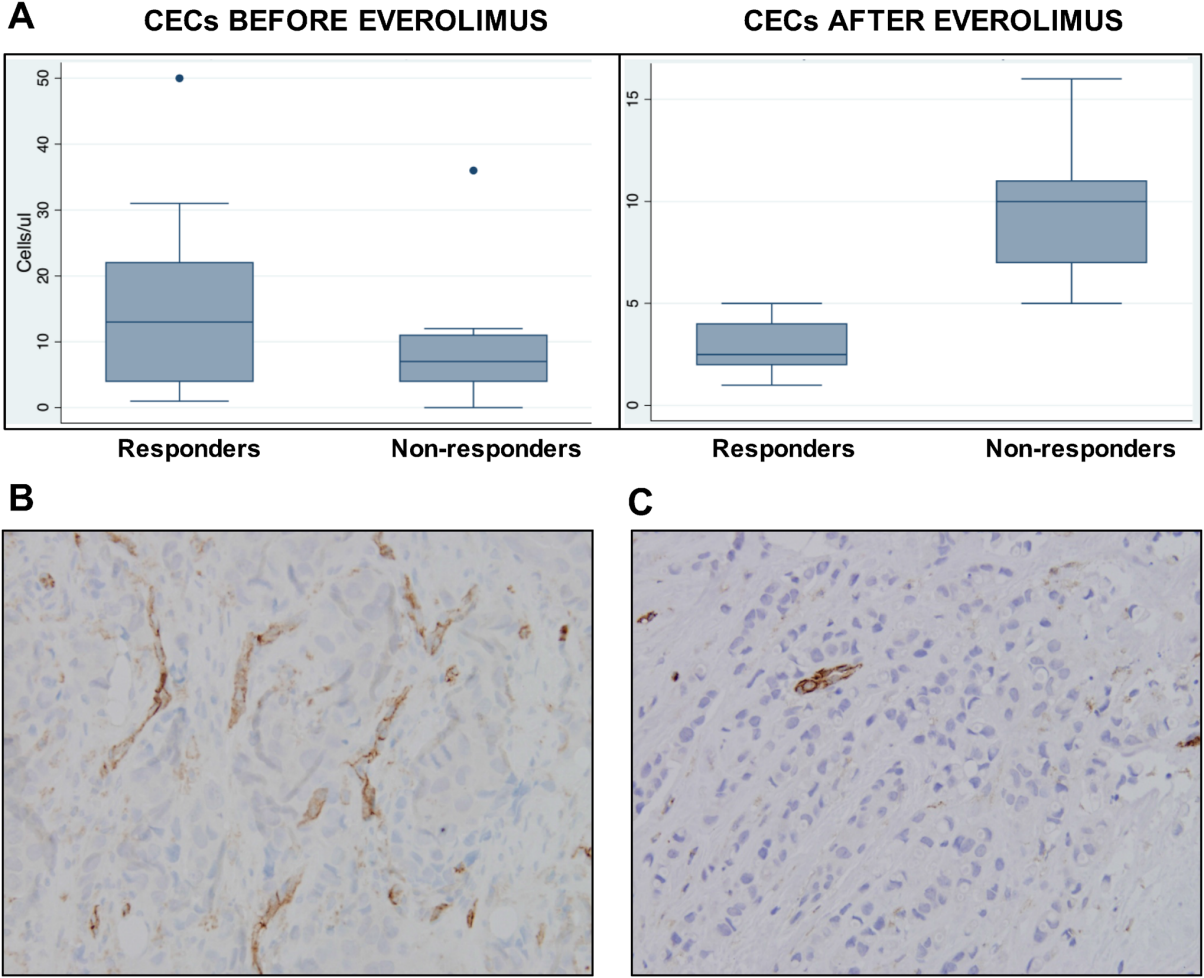
Quantification of CEC in blood of responsive and non-responsive patients before and after treatment with everolimus (**a**) and representative images of CD31 + vessels in tumor tissues of responsive (**b**) and non-responsive (**c**) patients

CECs were found in all 15 patients. No significant differences were observed between responders and non-responders before treatment (Fig. 3a). However, after everolimus treatment, there was a significant reduction in CECs number only in responders, resulting in a highly significant different numbers between responders and non-responders (*p* < 0.001), demonstrating the biological activity of everolimus. Notably, responders showed a higher tumor vascularisation at baseline using CD31+ vascular density (Fig. 3b), compared with non-responders (Fig. 3c).

### Prognostic significance of the NLR in the BALLET Study

**Fig. 4:**
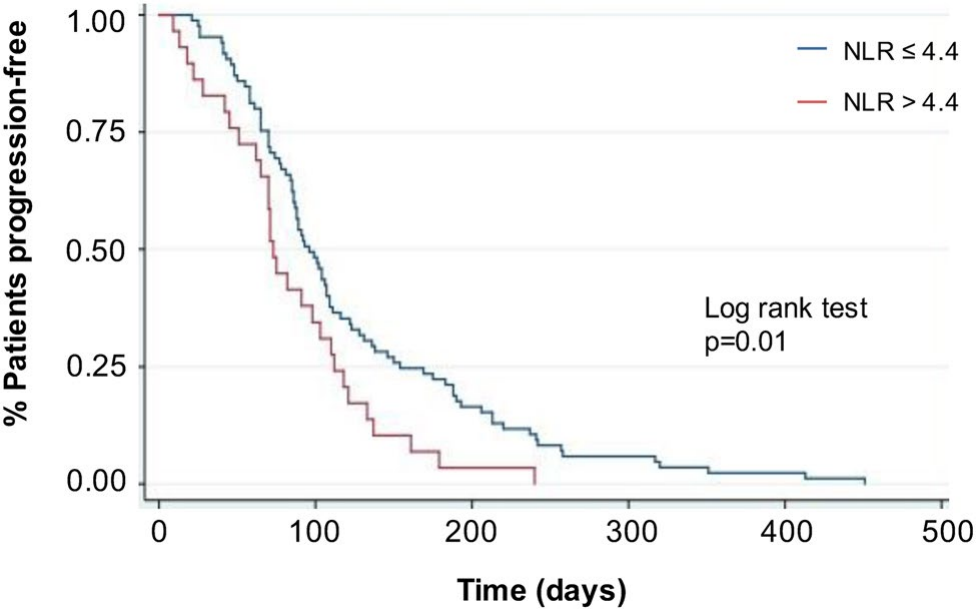
Kaplan–Meier curves of progression-free survival of patients with NLR ≤ 4.4 vs. NLR > 4.4 from the BALLET trial

Blood cell counts were obtained from 114 patients. The following NLR-based quartiles were generated: quartile 1 (NLR ≤ 2.3), quartile 2 (2.3 < NLR ≤ 3.2), quartile 3 (3.2 < NLR ≤ 4.4), , and quartile 4 (NLR > 4.4). As shown in Table 1, the median lymphocyte and neutrophil counts differed significantly among the 4 groups (*p* < 0.001 for both), without differences in basophils (*p* = 0.82), eosinophils (*p* = 0.63), monocytes (*p* = 0.21), and platelets (*p* = 0.32). The differences in PFS were analyzed through Kaplan–Meier curves and log-rank test. Overall, a statistically significant difference was observed when comparing all the 4 patient groups (*p* = 0.01). When comparing NLR ≤ 2.3 vs. NLR > 2.3 (*p* = 0.19), NLR ≤ 3.2 vs. NLR > 3.2 (*p* = 0.12), and NLR ≤ 4.4 vs. NLR > 4.4 (*p* = 0.01), the lower quartile was always apparently favored in terms of PFS, compared to the higher; however, a statistically significant difference was only observed when comparing NLR ≤ 4.4 vs. NLR > 4.4 (*p* = 0.01; Fig. 4). From each comparison, it was possible to evince that lower NLR corresponds to better survival outcomes in mBC treated with everolimus.

### Ingenuity pathway analysis according to response to everolimus in neoadjuvant setting

**Fig. 5:**
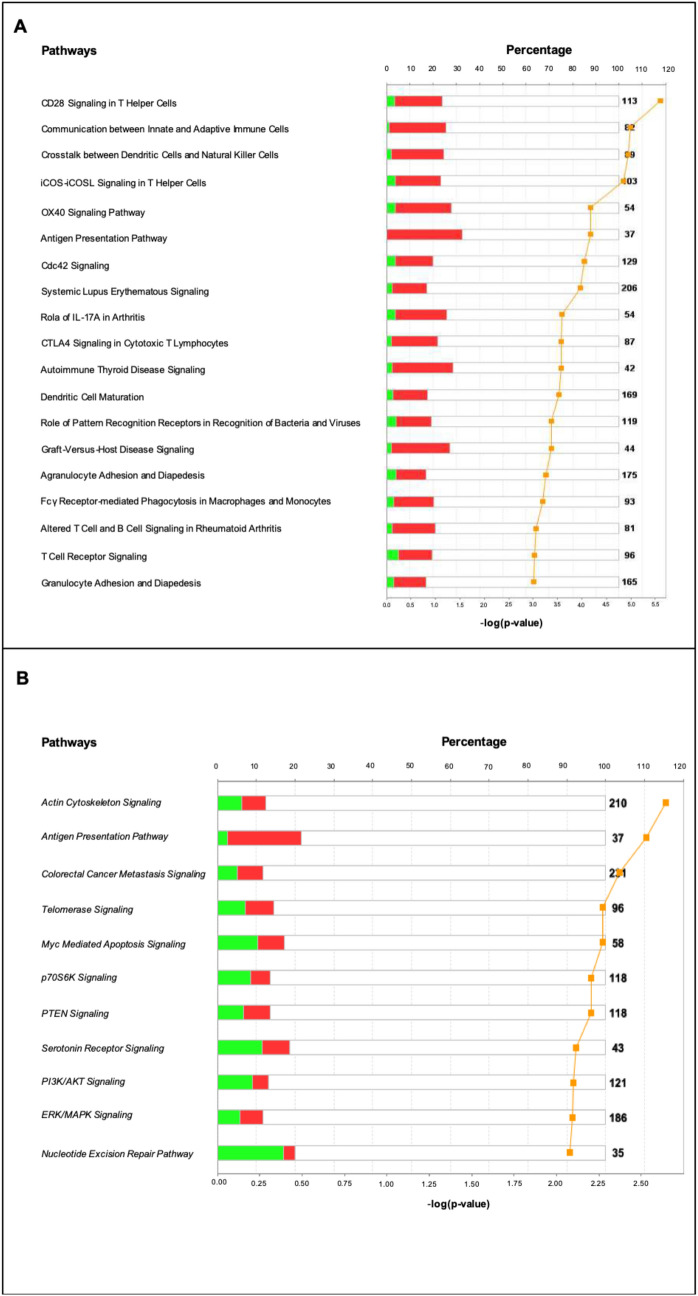
Gene classification according to canonical signaling pathways using Ingenuity Pathway Analysis (IPA), before (**a**) and after (**b**) everolimus treatment. The bars denote the percentage of downregulated (green) and upregulated (red) differentially expressed genes in responsive compared to non-responsive tumors out of the total number of genes present in the IPA database (shown in black to farthest right) within each pathway. Orange squares represent − log (*p* value)

Overall, 2063 genes were differentially expressed between everolimus “responders” and “non-responders” before treatment, as observed elsewhere [26]. Between the two groups, several pathways were found to be associated with the immune system, as top scoring (*p* < 0.001) (Fig. 5a), with the majority of innate and adaptive immunity-related genes up-modulated in everolimus-responsive compared with everolimus-unresponsive tumors before treatment. Post-treatment, the majority of pathways that were differentially enriched in responders compared with non-responders were those typically represented in epithelial cells and associated with response to everolimus, such as PI3K, actin cytoskeleton and ERK, with the majority of genes downregulated in responsive tumors (Fig. 5b). The only immune-related pathway that remained significantly positively enriched in responsive tumors was the one related to antigen presentation (Fig. 5b).

## Discussion

Everolimus with exemestane has been approved for the treatment of postmenopausal HR+/HER2− mBC following a significant PFS improvement observed in the BOLERO-2 trial [34]. After that, newer effective treatment strategies based on CDK4/6 inhibitors combined with aromatase inhibitors or fulvestrant and the PI3K inhibitor alpelisib combined with fulvestrant for PIK3CA-mutant patients have also been added to the therapeutic armamentarium in the last few years [35–37]. A recent comprehensive network meta-analysis highlighted comparable therapeutic performances between such therapies and chemotherapy [38]. However, at present, the optimal treatment sequence is not known, as there is a lack of direct comparisons and no effective biomarkers of response for all these treatment strategies.

This study was designed to identify potential biomarkers of response to everolimus, with the aim of better recognizing patients with a higher probability of benefiting from everolimus, and tend towards a more personalized treatment approach for HR+/HER2− mBC. Our study supports the notion that HR+ breast cancer patient’s responsiveness to everolimus, as described for other targeted therapies [6], might be mediated by an interplay with the immune system. Thus, an immune system biomarker could be a valuable tool to identify patients most likely to benefit from this drug.

The IPA showed that, before treatment, several pathways associated with active immune response were upregulated in everolimus responders compared to non-responders. Interestingly, everolimus treatment induced the loss of enriched immune pathways in responders, apart from those related to antigen presentation. Furthermore, NLRs in blood samples derived from the BALLET study showed that lower basal NLRs were associated with better PFS. More specifically, our analysis pointed out a significant difference only when comparing all the lowest quartiles to the highest. Of note, a recently published study, among various results, confirmed an unfavorable prognostic role for high levels of NLR in MBC, by using propensity score-matched MBC patients and healthy women [39].

NLRs have long been observed to be correlated with prognosis upon everolimus treatment also in other types of solid tumors (e.g., in patients with renal cell carcinoma treated with everolimus) [15]. Although the precise immune system’s microenvironment of RC is likely to be different from that in BC, our results from the BALLET study seem to support a common mechanism at the basis of everolimus anti-tumor activity, at least in patients with very high NLR values. Our results from the first large study of mBC patients treated with everolimus, although preliminary, suggest that a simple NLR might be a useful clinical tool without additional costs to determine everolimus responders a priori. Moreover, another study showed a better overall survival for patients with MBC and stable low NLR through time and treatment change [39]. This suggests that the evaluation of the dynamics of NLR might also be studied to understand its relevance in monitoring treatment efficacy.

Another potential biomarker is T lymphocyte subpopulation. Our analysis of everolimus-treated mBC patients within the mTOR Study showed that both at baseline and after everolimus treatment, overall T lymphocytes, including both CD8+ (T-killers) and T-helpers CD4+ were significantly higher in everolimus responders vs. non-responders with a trend for a Tregs CD4+ reduction, in keeping with the prognostically favorable role of lower NLR basal values observed in the BALLET patients. A higher number of T-helper might explain the higher number of CD8+ in everolimus responders, being the first particularly involved in recruiting and activating the effectors T-killers in immune adaptive responses. At the same time, a reduction of Tregs might be responsible for the increase in both T-helpers and killers, due to Tregs immunosuppressive function [40, 41] supported by preclinical studies of murine tumor models [42]. Albeit speculative, it is possible that patients with higher infiltration of these cells in the tumor tissue before treatment are those that better benefit from treatment, due to the presence of the cell targets of everolimus. In fact, mTOR is active in immune cells, where it regulates important and diverse functions in all T-cell lineages [43]. Nevertheless, the Tregs reduction was not statistically significant and the number of patients was too small to draw any definitive conclusion.

The high pre-treatment infiltration of immune cells in responsive tumors might mirror their high intrinsic basal mTOR activation, reported to be involved in the recruitment of immune cell in the tumor microenvironment [44]. Everolimus on-target activity in these tumors could thus explain the downregulation of immune pathways after treatment in everolimus responders and consequent lack of differences in immune pathways with non-responders observed after treatment. In accordance with this, hypothesis is also the association between low number of CECs and response in patients on treatment with everolimus. Indeed, the levels of CECs, a potential neoangiogenesis marker [20], correlate with plasma levels of VCAM-1 and VEGF [19], whose downstream pathways include PI3K/Akt/mTOR signaling and are also inhibited by everolimus [45]. In this context, the higher basal vascularity in tumor tissues in responders, compared to non-responders, might reflect the higher activation of the mTOR pathway in tumors from patients who will benefit the most from everolimus treatment. Thus, the reduction in circulating CECs in patients on treatment with everolimus might represent a potential midcourse biomarker for guiding patients toward the ideal regimen after brief exposure to everolimus.

We are aware that this work has several limitations. First of all, the retrospective nature of the three studies limits the statistical power and the number of variables analyzed, such as time-to-drug exposure, at the decision of the investigators. Secondly, the total number of patients analyzed in the local study (15 patients) and the neoadjuvant study (23 patients) is relatively small and different kinds of analyses were conducted on the different cohorts of patients. Moreover, the cohorts of the studies differ in terms of clinical setting (neoadjuvant vs metastatic) and none of the studies included a control arm, needed to clearly distinguish between a prognostic and a predictive role.

However, the importance of our study relies in the facts that, to our knowledge, for the first time, the potential relevance of lymphocytes subpopulations, CECs, and NLR as easily-detectable biomarkers of response to everolimus-based regimens in HR+ BC is reported.

Despite not being conclusive, our data, corroborated by an increasing body of evidence [39, 46], might provide the rationale for larger, prospective and more homogeneous trials, which could pave the way to the development of a new tool capable of easily predicting and monitoring everolimus response in HR+/HER2− BC.

**Table 1:** Blood cells count according to NLR quartiles

Blood cell line^a^	Quartile 1	Quartile 2	Quartile 3	Quartile 4	No. of patients	*p*^*#*^
	NLR ≤ 2.3	2.3 < NLR ≤ 3.2	3.2 < NLR ≤ 4.4	NLR > 4.4		
Monocytes					114	0.21
Median	0.44	0.58	0.53	0.65		
Min–max range	0.14–1.31	0.2–1.76	0.2–1.22	0.15–6.6		
Lymphocytes					114	** < 0.01**
Median	2	1.51	1.36	0.82		
Min–max range	0.76–4.74	0.83–4.69	0.74–1.93	0.36–16.7		
Neutrophils					114	** < 0.01**
Median	3	4.23	4.89	5.73		
Min–max range	1.24–8.91	1.9–12.79	2.68–8.43	3.12–74		
Basophils					113	0.82
Median	0.02	0.02	0.03	0.02		
Min–max range	0–0.2	0–0.19	0–0.11	0–0.6		
Eosinophils					113	0.63
Median	0.1	0.08	0.07	0.09		
Min–max range	0–0.58	0–0.35	0–0.32	0–2		
Platelets					114	0.32
Median	213	240	267	261		
Min–max range	56–442	160–445	92–517	118–636		

*NLR* neutrophils-to-lymphocytes ratio

^a^Cells × 10^3^/mL

^#^Kruskal–Wallis test for continues variables
